# ATP/P2X7 signaling as a potential predictive biomarker for early recurrence of nasal polyps: Correlation with inflammasome activation and epithelial barrier dysfunction^☆^

**DOI:** 10.1016/j.waojou.2026.101455

**Published:** 2026-08-28

**Authors:** Linfei Yang, Yi Chen, Ting Shi, Qiong Liu

**Affiliations:** a Department of Clinical Laboratory, Hunan Provincial People's Hospital (The First-Affiliated Hospital of Hunan Normal University), Changsha, Hunan Province, 410005, China; b Department of Otorhinolaryngology-Head and Neck Surgery, Hunan Provincial People's Hospital (The First-Affiliated Hospital of Hunan Normal University), Changsha, Hunan Province, 410005, China

**Keywords:** Chronic rhinosinusitis with nasal polyps, Postoperative recurrence, P2X7, Epithelial barrier, Biomarker

## Abstract

**Background:**

Chronic rhinosinusitis with nasal polyps (CRSwNP) is characterized by pronounced epithelial remodeling and barrier dysfunction, contributing to a high rate of postoperative recurrence. However, molecular mechanisms associated with early recurrence remain poorly understood.

**Methods:**

We performed paired proteomic profiling of nasal polyp tissues obtained from the same patients before initial surgery and at the time of recurrence (discovery cohort) to identify recurrence-associated proteins. Candidate proteins were validated in an independent cohort, and their association with recurrence risk was assessed by receiver operating characteristic (ROC) curve and Kaplan-Meier analyses. The expression and cellular localization of the identified candidate were further characterized by immunofluorescence. Mechanistic investigations were conducted in primary human nasal epithelial cells using ATP stimulation and specific pharmacological inhibitors.

**Results:**

Paired proteomic analysis identified P2X7, PDE4D, and JADE2 as the top 3 upregulated proteins in recurrent tissues. Among these, only P2X7 was consistently elevated in the validation cohort and predominantly localized to the nasal epithelium (P < 0.01). ROC curve analysis indicated that tissue P2X7 level might serve as a potential predictive marker for postoperative recurrence of CRSwNP. (AUC = 0.678, P = 0.022). Kaplan-Meier analysis further showed that the risk of recurrence was significantly higher in the high P2X7 expression group (HR = 2.323, P = 0.015). Recurrent tissues exhibited elevated levels of NLRP3 and IL-1β, reduced expression of epithelial barrier markers occludin and E-cadherin, accompanied by increased extracellular ATP concentrations. In vitro, ATP stimulation upregulated P2X7 expression, induced NLRP3 and cleaved IL-1β, and downregulated occludin and E-cadherin. Notably, these effects were reversed by a P2X7-specific inhibitor (A-438079).

**Conclusion:**

Paired proteomic profiling revealed distinct protein expression profiles between pre- and post-recurrence nasal polyps, with P2X7 serving as a promising predictive biomarker for early recurrence. Mechanistically, the ATP/P2X7 axis may drive disease recurrence by potentially promoting inflammasome activation and epithelial barrier disruption.

## Introduction

Chronic rhinosinusitis with nasal polyps (CRSwNP) is a highly heterogeneous inflammatory disease of the sinonasal mucosa.^1^^,^^2^ Although functional endoscopic sinus surgery (FESS) remains a primary treatment modality, a substantial proportion of patients experience postoperative recurrence.^3^^,^^4^ Previous studies have found that the recurrence rate following surgery for CRSwNP may range from 40% to as high as 75% more than a decade later,^5^, ^6^, ^7^ which significantly impacts their quality of life and poses a major challenge in clinical practice. Although the pathogenesis of CRSwNP is recognized to involve a complex interplay of type 2 (T2) inflammation and defective epithelial barrier restitution,^8^, ^9^, ^10^ the specific molecular drivers that predispose patients to refractory relapse remain poorly understood. Therefore, there is an urgent clinical need for reliable prognostic biomarkers to enable early identification of patients at high risk of recurrence.

Although previous investigations have utilized transcriptomic, metabolomic, and microbiome profiling to explore recurrence-associated biomarkers in CRSwNP,^11^^,^^12^ these approaches offer complementary insights rather than directly capturing the functional endpoints of disease pathology. Given that proteins directly perform cellular functions, proteomic analysis provides a more direct assessment of tissue remodeling and immune dysregulation.^13^^,^^14^ However, tissue-based proteomic studies focusing on recurrent CRSwNP remain scarce. To our knowledge, systematic characterization of proteomic alterations specifically in paired pre- and post-recurrence tissues from the same patients has not been reported, leaving a critical gap in understanding the molecular events driving early relapse.

Therefore, the present study was designed to characterize the proteomic alterations specifically associated with early recurrence in CRSwNP using paired tissue samples obtained before and after recurrence from the same patients. By performing paired proteomic profiling, we aimed to identify differentially expressed proteins linked to disease relapse. Candidate proteins were subsequently validated in an independent cohort, and the functional role of the key molecule was further investigated through *in vitro* experiments to elucidate the underlying mechanisms driving recurrence.

## Materials and methods

### Study population and tissue collection

This study was approved by the Human Ethics Committee of Hunan Provincial People's Hospital (The First-Affiliated Hospital of Hunan Normal University) (Approval No.: 2026036), and all participants provided written informed consent in accordance with the Declaration of Helsinki. Informed consent was obtained from all participants prior to include. In order to determine the protein markers related to recurrence, nasal polyps of the 8 same patients who underwent primary surgery and revision surgery were collected. Subsequently, an independent validation cohort was established, comprising controls (n = 30) (histologically normal uncinate mucosa from patients with benign skull base tumors), non-recurrent CRSwNP patients (n = 38), and recurrent CRSwNP patients (n = 22). The diagnosis of CRSwNP was made by experienced otolaryngologists based on EPOS2020 criteria.^15^ All enrolled patients, who had failed to respond to at least 12 weeks of maximum medical therapy, underwent functional endoscopic sinus surgery (FESS) in strict accordance with established international guidelines, and all were scheduled for preoperative nasal endoscopy and sinus CT scans. All endoscopic examinations and subsequent scoring were performed independently by 2 experienced senior otolaryngologists. Nasal endoscopy confirmed the presence of bilateral nasal polyps, while CT scans assessed the extent of sinus involvement. Patients with immunodeficiency, cystic fibrosis, aspirin-exacerbated respiratory disease, or recent use of systemic corticosteroids/biologics within 4 weeks prior to surgery were excluded.

### Preoperative medical treatment and definition of recurrence

Before surgery, all included patients received at least 12 weeks of standard, optimized, maximum-dose medical therapy as per treatment guidelines. This consisted of daily intranasal corticosteroids (mometasone furoate nasal spray 200 μg per nostril once daily, total 400 μg/day) and twice-daily nasal saline irrigation (20 mL per nostril with 0.9% sodium chloride). All CRSwNP patients received FESS from 3 senior surgeons under uniform standards. Surgical extent was guided by preoperative CT and intraoperative findings. The main objective was to restore ventilation and mucociliary clearance by precisely removing lesions while preserving healthy mucosa, thereby avoiding ostial stenosis and reducing surgical confounding in recurrence analysis. After surgery, all patients with CRSwNP were followed up for a minimum of 1 year. Clinical recurrence is defined as: endoscopic or imaging (CT) examination shows that polyps reappear, and despite receiving standard drug treatment, intractable symptoms persist for more than 8 weeks.^16^^,^^17^ According to these diagnostic criteria, the cohort was then divided into recurrent group and non-recurrent group.

### Tissue preparation and proteomic profiling

Nasal polyp and middle turbinate mucosal tissues were immediately cryopreserved in liquid nitrogen following surgical resection. The frozen specimens were ground into fine powder under cryogenic conditions and then lysed in RIPA buffer supplemented with a protease inhibitor cocktail to prevent protein degradation. After high-speed centrifugation, the supernatant containing soluble proteins was collected. Protein concentrations were determined using the bicinchoninic acid (BCA) assay to ensure equal protein input for subsequent steps. Equivalent amounts of protein from each sample were subjected to reduction and alkylation to reduce and block disulfide bonds, followed by overnight digestion at 37 °C using trypsin (sequencing grade). The resulting peptide mixtures were desalted by solid-phase extraction and concentrated under vacuum. Peptide separation was performed via reverse-phase high-performance liquid chromatography (RP-HPLC) on a C18 column with a predefined acetonitrile gradient. The eluted peptides were analyzed by liquid chromatography-tandem mass spectrometry (LC-MS/MS) operated in data-dependent acquisition (DDA) mode to achieve broad and reliable peptide identification.

### Bioinformatics analysis

Raw LC-MS/MS data were processed using MaxQuant for label-free quantification and protein identification against the human UniProt/SwissProt reference proteome. Proteins detected in at least 70% of biological replicates within any given group were retained for further analysis. Differentially expressed proteins (DEPs) were identified using Perseus software, with thresholds set at a fold change >1.5 and a p-value <0.05 as previously described.^12^^,^^18^

### Tissue ATP quantification

ATP levels in nasal polyp and mucosal tissues were measured using a commercial ATP Assay Kit (Cat. No. S0026, Beyotime Biotechnology, Shanghai, China) according to the manufacturer's instructions. Briefly, frozen tissue samples were weighed and homogenized in the provided lysis buffer (100–200 μL per 20 mg tissue) using a glass homogenizer to achieve complete cell lysis. The homogenates were centrifuged at 12,000×*g* for 5 min at 4 °C, and the supernatants were collected. The cleared lysates were then mixed with ATP detection reagent containing firefly luciferase and luciferin, and luminescence was immediately measured using a microplate luminometer. ATP concentrations were normalized to total protein content determined by a standard protein assay.

### Primary nasal epithelial cell culture and treatments

Human nasal epithelial cells (NECs) were isolated from middle turbinate mucosal tissues of control subjects as previously described.^19^^,^^20^ Briefly, fresh tissues were rinsed with PBS to remove mucus and blood, cut into small pieces, and digested with a collagenase-trypsin mixture at 37 °C for 30–60 min. The digestion was neutralized with FBS-containing medium, and the cell suspension was filtered through a sterile cell strainer. After centrifugation at 300×*g* for 5 min, the pelleted cells were resuspended in PneumaCult™-Ex Plus medium (STEMCELL Technologies, Vancouver, Canada) and cultured in a humidified incubator (37 °C, 5% CO_2_). Upon reaching 70–80% confluence, primary NECs were passaged into 6-well plates at a density of 1 × 10^5^ cells/well. When cultures reached approximately 80% confluence, they were subjected to the indicated treatments. To establish an ATP-induced injury model, NECs were stimulated with ATP (100 μM) for specified durations.^21^ For antagonist rescue experiments, cells were pre-incubated with the selective P2X7 antagonist A-438079 (10 μM) for 1 h prior to ATP or BzATP.^22^

### RNA extraction and quantitative real-time PCR (RT-qPCR)

Liquid nitrogen-frozen tissues were lysed in TRIzol reagent, followed by standard chloroform extraction and isopropanol precipitation. The purified RNA pellets were washed with 75% ethanol, dissolved in RNase-free water, and quantified via spectrophotometry. Subsequently, reverse transcription was carried out using the PrimeScript RT Master Mix to generate cDNA. Quantitative PCR amplification was performed using SYBR Premix EX Taq (Takara, Dalian, China). The relative expression of target transcripts was calculated based on the 2^−ΔΔCt^ method and normalized against GAPDH. Primer sequences used in this study are listed in Supplementary Table S1.

### Western blotting (WB)

Protein lysates were separated by SDS-PAGE and transferred onto PVDF membranes. After blocking with 5% non-fat milk, the membranes were incubated overnight at 4 °C with primary antibodies against NLRP3 (DF7438, 1:1000), Cleaved-IL-1β (AF4006,1:500), E-cad (BF0219, 1:500), Occludin (DF7504, 1:500) and beta-actin (AF7018, 1:500), all supplied by Affinity Biosciences (Changzhou, China). Following incubation with HRP-conjugated secondary antibodies, protein bands were visualized using an enhanced chemiluminescence (ECL) detection system and quantified by ImageJ software (NIH, Bethesda, USA).

### Tissue and cell immunofluorescence (IF)

Tissue sections (5 μm) were prepared from paraffin-embedded samples fixed in 4% paraformaldehyde (PFA). After deparaffinization and rehydration, antigen retrieval was performed using microwave heating. For cultured cells, nasal epithelial cells grown on coverslips were fixed with 4% PFA for 20 min and permeabilized with 0.2% Triton X-100 for 10 min at room temperature. Both tissue sections and cell coverslips were then blocked with 5% bovine serum albumin (BSA) for 1 h (0.3% Triton X-100 was added to the blocking buffer for tissues). Samples were incubated overnight at 4 °C with primary antibodies against P2X7 (BF8403, 1:100), NLRP3 (DF7438, 1:100), IL-1β (DF7438, 1:100), E-cad (BF0219, 1:100), or occluding (DF7504, 1:100), all supplied by Affinity Biosciences (Changzhou, China). After washing, they were incubated with fluorophore-conjugated secondary antibodies for 1 h at room temperature in the dark. In addition, to eliminate background signals caused by nonspecific antibody binding during immunofluorescence staining, an isotype control was included in this study. Under the same conditions as those used for the primary antibody, a rabbit IgG isotype control antibody was used in place of the primary antibody for incubation; all subsequent steps were performed in the same manner as for the experimental group. Nuclei were counterstained with DAPI, and slides were mounted with antifade medium. Fluorescence images were captured using a fluorescence microscope, and the fluorescence intensity or percentage of positive cells was analyzed using ImageJ software (NIH, Bethesda, USA).

### Statistical analysis

Statistical analyses were performed using GraphPad Prism 8.3 (GraphPad Software, San Diego, SA) and SPSS 25.0 (IBM Corp., Armonk, USA). Continuous variables were evaluated for normal distribution utilizing the Shapiro-Wilk test. Normally distributed data were expressed as mean ± standard deviation (SD), whereas non-normally distributed data were presented as medians with interquartile ranges. Due to the small sample size in the discovery cohort, non-parametric tests were strictly employed to avoid bias; comparisons between 2 paired groups were analyzed using the Wilcoxon signed-rank test, while comparisons among 3 groups in the validation cohort were assessed using one-way analysis of variance (ANOVA). Categorical variables were analyzed using the chi-square test. Correlation analyses were performed using Pearson's or Spearman's correlation coefficients, depending on the data distribution. To assess the predictive value of P2X7 levels for recurrence in CRSwNP, receiver operating characteristic (ROC) curves were plotted and the area under the curve (AUC) was calculated. Kaplan-Meier curves were used to depict the time to recurrence, and intergroup differences were compared using the log-rank test. A two-sided P-value <0.05 was considered statistically significant.

## Results

### Paired proteomic analysis reveals distinct protein expression profiles between pre- and post-recurrence nasal polyps

**Fig. 1:**
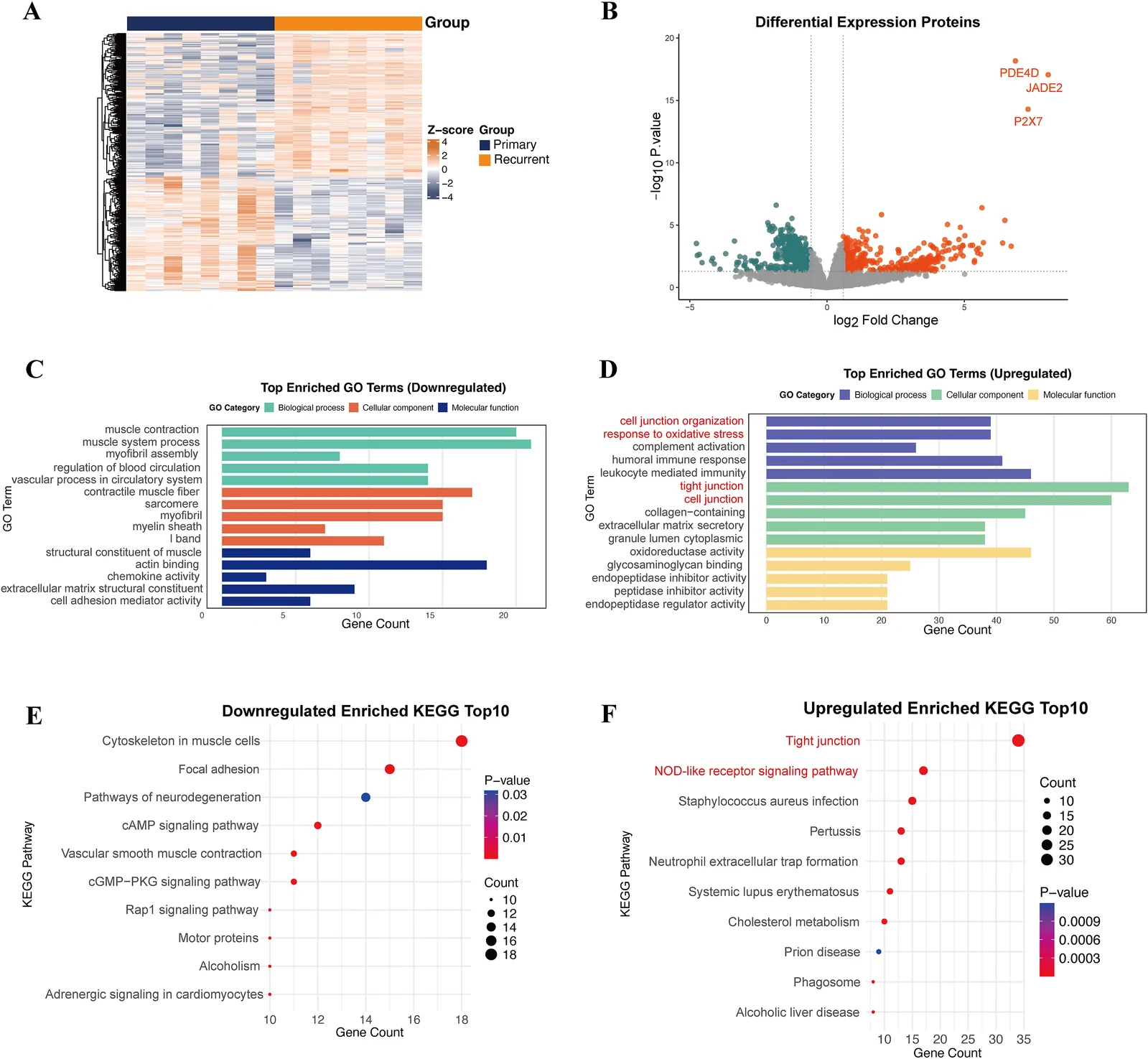
**Proteomic analysis of Primary and Recurrent CRSwNP tissues. (A)** Heatmap of differentially expressed proteins showing distinct clustering between Primary and Recurrent groups. **(B)** Volcano plot highlighting significantly upregulated proteins. **(C**–**D)** Top enriched GO terms for downregulated and upregulated proteins, featuring key processes like response to oxidative stress and tight junction. **(E**–**F)** Top 10 enriched KEGG pathways for downregulated and upregulated proteins. CRSwNP, Chronic rhinosinusitis with nasal polyps; GO, Gene Ontology; KEGG, Kyoto Encyclopedia of Genes and Genomes

To investigate the molecular mechanisms of disease recurrence, we performed paired proteomic analysis on primary and recurrent tissue specimens obtained from the same patients. The clinical characteristics of the discovery cohort are detailed in Table 1. Unsupervised hierarchical clustering revealed distinct global protein expression profiles distinguishing the recurrent group from the primary group (Fig. 1A). Volcano plot analysis identified P2X7, PDE4D, and JADE2 as the most significantly upregulated proteins in recurrent tissues (Fig. 1B). Functional enrichment analysis by Gene Ontology (GO) and Kyoto Encyclopedia of Genes and Genomes (KEGG) indicated that DEPs were predominantly associated with structural and contractile processes, including muscle contraction and focal adhesion, whereas upregulated proteins were closely linked to pathways implicated in epithelial barrier dysfunction, oxidative stress, and inflammatory immune responses, including tight junction and NOD-like receptor signaling (Fig. 1C–F). Collectively, these findings demonstrate substantial proteomic differences between pre- and post-recurrence nasal polyps, with P2X7, PDE4D, and JADE2 representing the most prominently upregulated molecules among a broader set of recurrence-associated protein alterations.

**Table 1:** Characteristics of patients in the discovery cohort

Variables	Primary group	Recurrent group	P-value
Sex, male/Female	5/3	5/3	1.000
Age, years	36.8 ± 4.9	37.8 ± 4.9	0.691
BMI, kg/m^2^	22.4 ± 3.5	24.7 ± 4.8	0.285
Allergic rhinitis, n (%)	2 (25.0)	2 (25.0)	1.000
Asthma, n (%)	1 (12.5)	1 (12.5)	1.000
Blood eosinophil counts, × 10^9^/L	0.2 ± 0.2	0.3 ± 0.2	0.444
Blood eosinophil percentage, %	3.6 ± 3.9	4.5 ± 3.2	0.630
Lund-kennedy score	7.8 ± 2.3	8.4 ± 1.9	0.560
Lund-mackay score	13.1 ± 3.2	13.9 ± 3.7	0.671
Disease duration, months	37.9 ± 5.5	49.9 ± 5.5	<0.001

### Validation of recurrence-associated proteins identifies P2X7 as a key predictor of postoperative recurrence

**Fig. 2:**
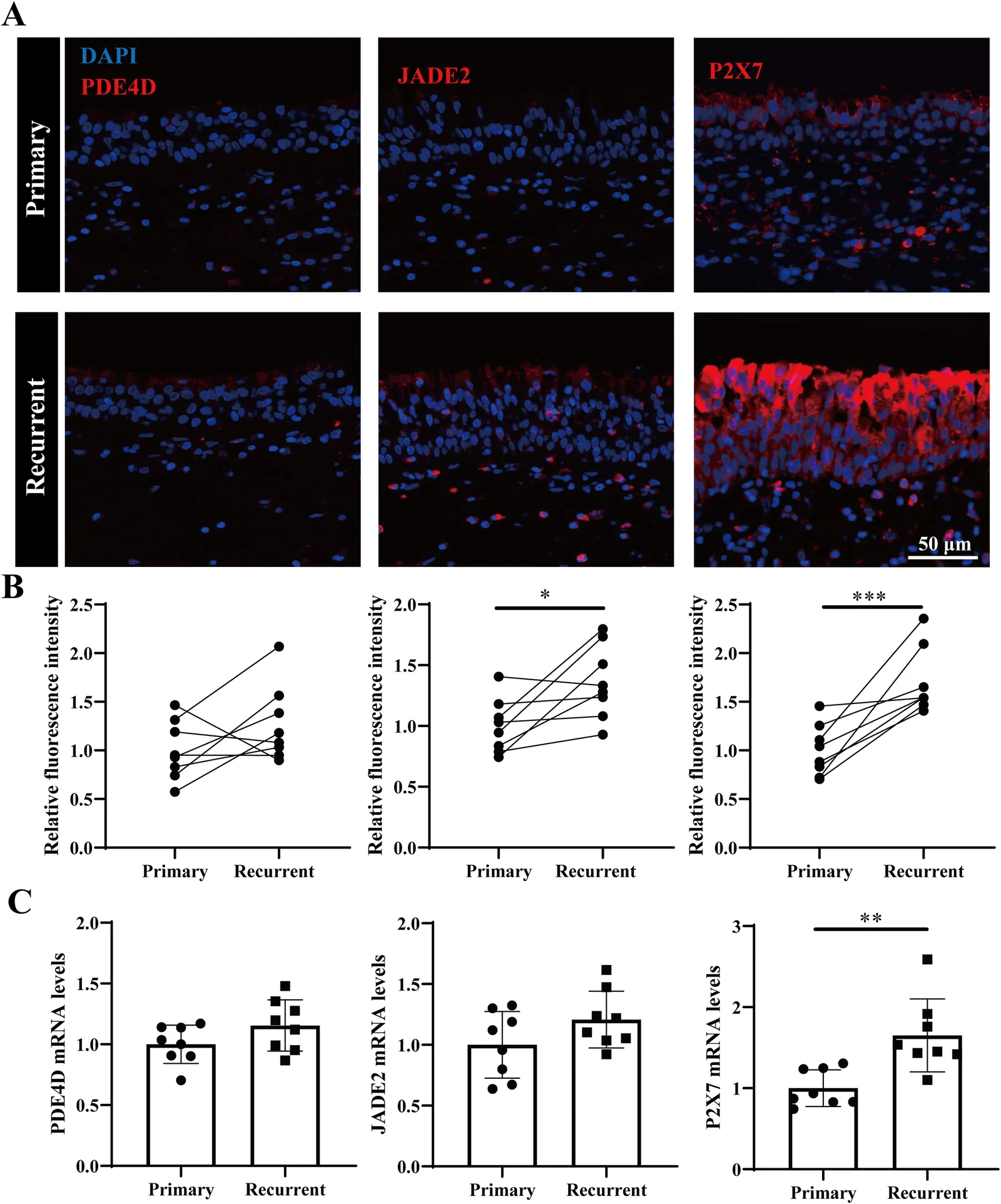
**Experimental validation of the top upregulated proteomic candidates. (A)** Representative immunofluorescence images of PDE4D, JADE2, and P2X7 in primary and recurrent tissue sections. **(B)** Quantification of relative fluorescence intensity for PDE4D, JADE2, and P2X7. **(C)** Relative mRNA expression levels of PDE4D, JADE2, and P2X7 determined by RT-qPCR. ∗P < 0.05, ∗∗P < 0.01, ∗∗∗P < 0.001. RT-qPCR, quantitative reverse transcription polymerase chain reaction

**Fig. 3:**
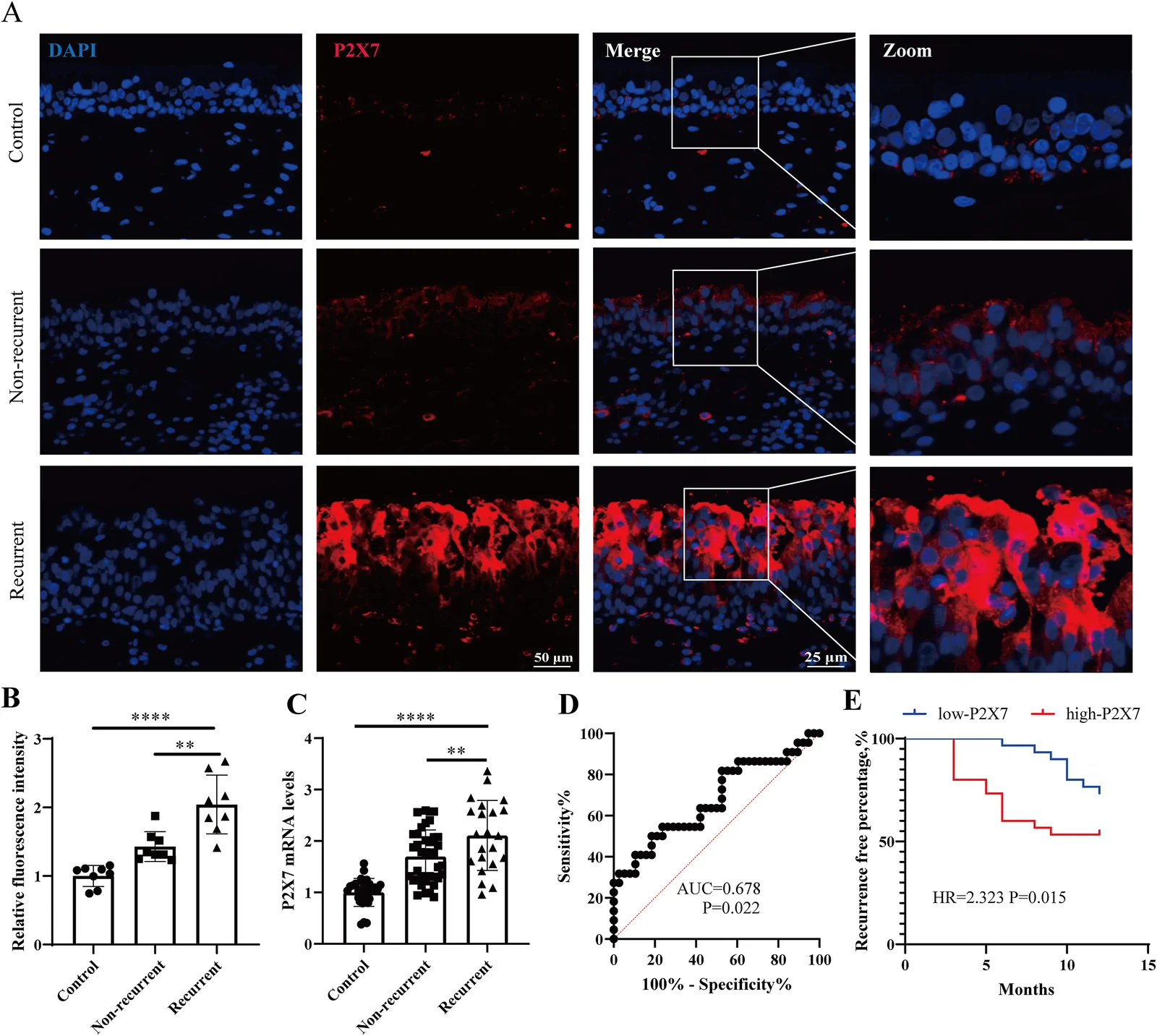
**P2X7 is upregulated in recurrent CRSwNP and serves as a prognostic biomarker for postoperative relapse.** (A) RT-qPCR analysis of P2X7 mRNA levels in control, primary, and recurrent tissue samples. (B) Representative IF images demonstrating P2X7 protein localization and abundance across the 3 clinical groups. (C) Quantification of relative P2X7 fluorescence intensity across the indicated groups. (D) ROC curve analysis evaluating the predictive efficacy of P2X7 for CRSwNP recurrence. (E) Kaplan-Meier analysis of recurrence-free survival in CRSwNP patients. ∗∗P < 0.01, ∗∗∗∗P < 0.0001. CRSwNP, chronic rhinosinusitis with nasal polyps; RT-qPCR, quantitative reverse transcription polymerase chain reaction; ROC, Receiver operating characteristic; HR, Hazard ratio

To validate the proteomic findings, we first examined the expression of the top 3 upregulated candidates using the same paired samples from the discovery cohort. Immunofluorescence staining revealed that both P2X7 and JADE2 were overexpressed in recurrent tissues compared with their matched primary counterparts, with predominant localization to the mucosal epithelium (Fig. 2A–B). Isotype controls are shown in Fig. S1. RT-qPCR analysis demonstrated that only P2X7 mRNA levels were significantly elevated in the recurrent group, whereas JADE2 and PDE4D showed no statistically significant differences (Fig. 2C). To further corroborate these findings, we assessed P2X7 expression level in a prospective validation cohort comprising 60 CRSwNP patients and 30 controls. Among these 60 patients, 22 experienced postoperative recurrence during a minimum follow-up of 1 year, while 38 remained recurrence-free (clinical characteristics summarized in Table 2). Both RT-qPCR and immunofluorescence staining analyses confirmed that P2X7 expression was significantly higher in the recurrent group compared with both non-recurrent patients and controls, with consistent epithelial localization (Fig. 3A–C). ROC analysis demonstrated that P2X7 mRNA levels possessed predictive value for early postoperative recurrence (AUC = 0.678, P = 0.022, Fig. 3D). Furthermore, Kaplan-Meier survival analysis revealed that patients with high P2X7 expression exhibited significantly lower recurrence-free survival rates compared with those with low expression (HR = 2.323, P = 0.015, Fig. 3E).

**Table 2:** Characteristics of patients in the validation cohort.

Variables	Control	Non-recurrent	Recurrent	P-value
Sex, male/Female	16/14	22/16	12/10	0.926
Age, years	38.2 ± 6.4	42.8 ± 11.1	41.6 ± 12.8	0.180
BMI, kg/m^2^	21.8 ± 2.0	22.1 ± 2.3	22.0 ± 2.6	0.838
Allergic rhinitis, n (%)	–	6 (15.8)	7 (31.8)	0.146
Asthma, n (%)	–	4 (10.5)	4 (18.2)	0.488
Blood eosinophil counts, × 10^9^/L	0.2 ± 0.1	0.2 ± 0.2	0.2 ± 0.2	0.882
Blood eosinophil percentage, %	3.6 ± 2.9	3.8 ± 2.4	3.8 ± 2.1	0.955
Lund-kennedy score	–	8.3 ± 1.6	8.7 ± 1.5	0.378
Lund-mackay score	–	13.4 ± 3.9	13.7 ± 3.6	0.727
Disease duration, months	–	28.4 ± 7.8	29.5 ± 6.6	0.604

BMI, Body mass index

### P2X7 upregulation correlates with inflammasome activation and barrier disruption in recurrent CRSwNP

**Fig. 4:**
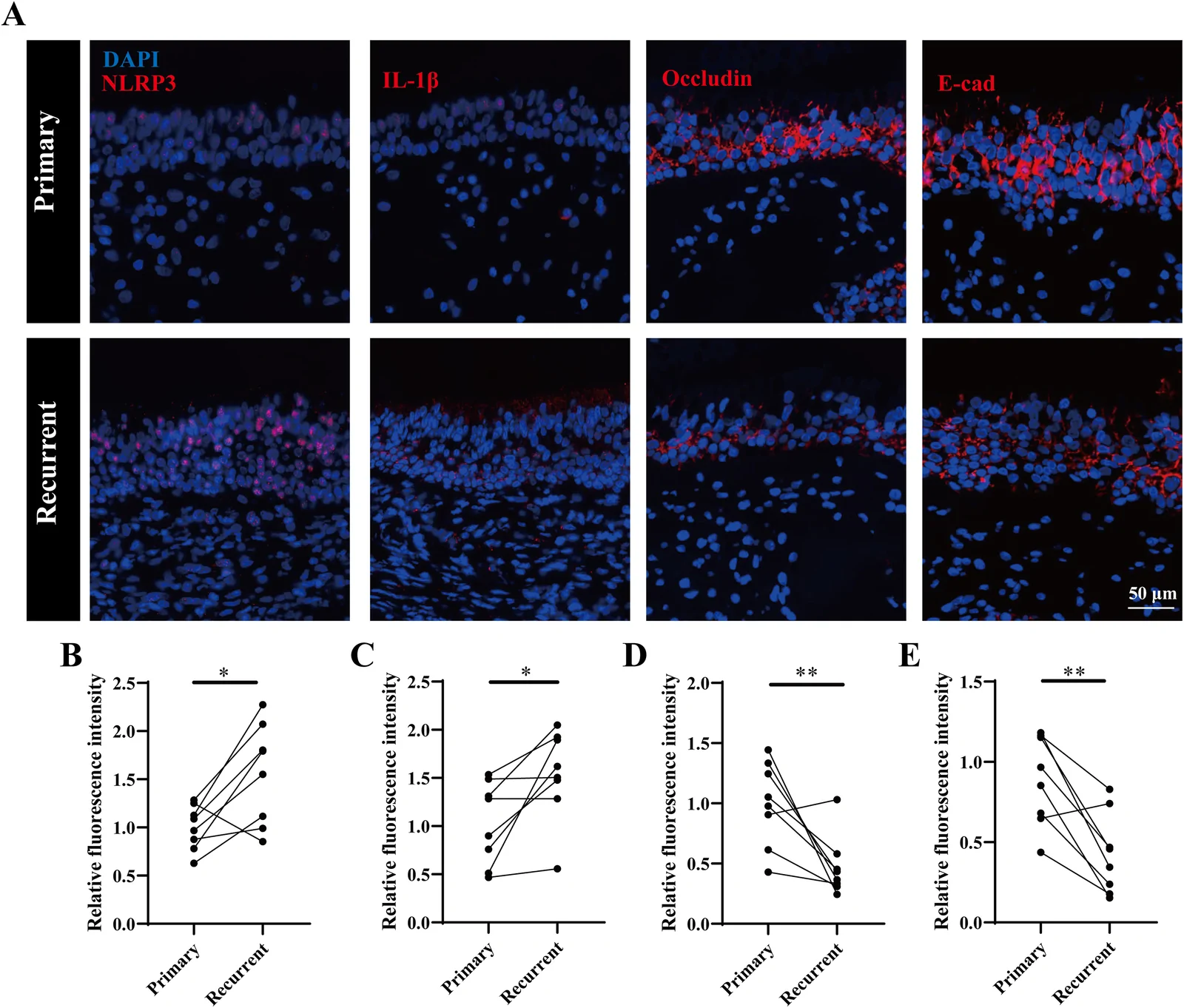
IF analysis of inflammasome markers and barrier proteins in discovery cohort. (A) Representative immunofluorescence images of NLRP3, IL-1β, occludin, and E-cad in primary and recurrent patients. (B–E) Quantitative analysis of relative fluorescence intensities for NLRP3, IL-1β, occludin, and E-cad. ∗P < 0.05, ∗∗P < 0.01. IF, Immunofluorescence, E-cad, E-cadherin.

**Fig. 5:**
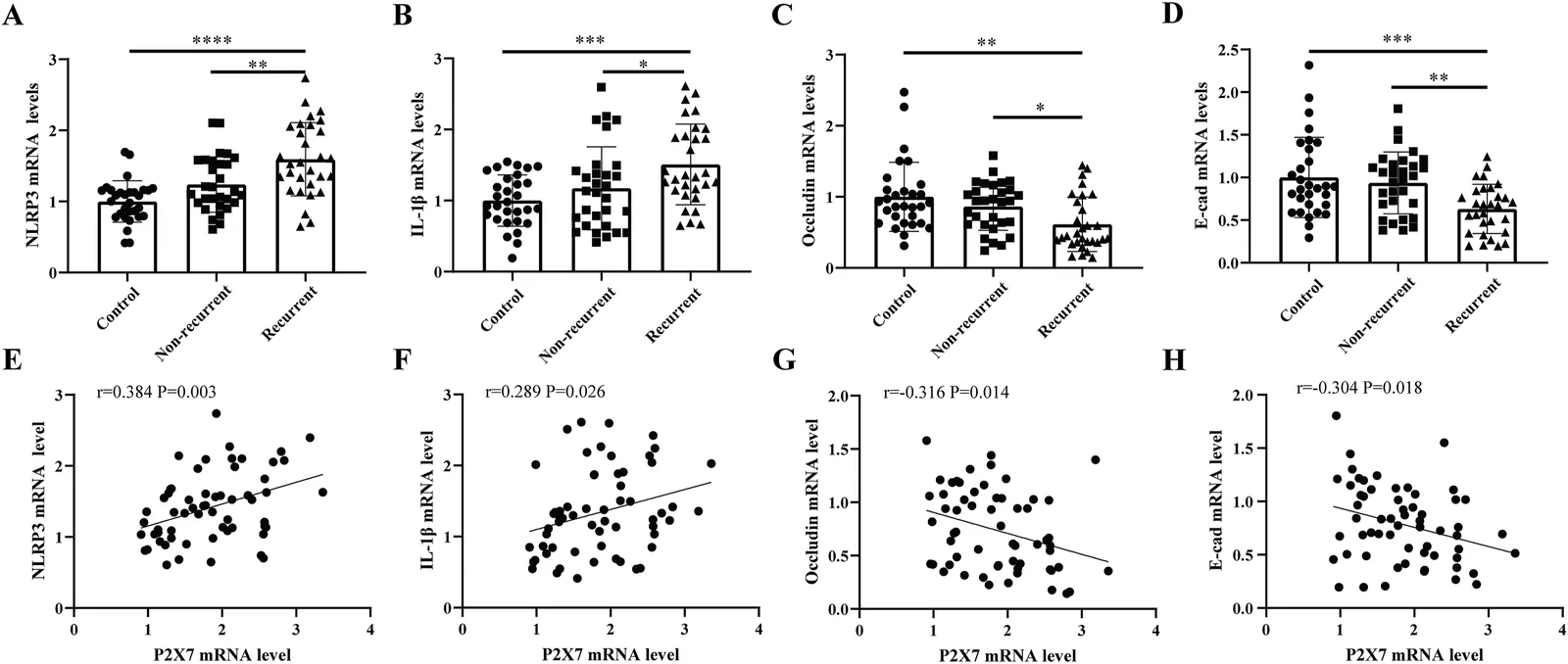
**P2X7 expression correlates with inflammasome markers and epithelial barrier disruption. (A**–**D)** qRT-PCR analysis of mRNA levels for the inflammasome components NLRP3 (A) and IL-1β (B), and the epithelial barrier proteins occludin (C) and E-cad (D) across control, non-recurrent, and recurrent group. **(E**–**H)** Pearson correlation between relative P2X7 mRNA levels and the expression of NLRP3, IL-1β, occludin, and E-cad. ∗P < 0.05, ∗∗P < 0.01, ∗∗∗P < 0.001, ∗∗∗∗P < 0.0001. RT-qPCR, quantitative reverse transcription polymerase chain reaction; E-cad, E-cadherin.

To explore the functional relevance of P2X7 upregulation, we examined the expression of NOD-like receptor signaling pathway components and tight junction markers in the discovery cohort, guided by the GO and KEGG enrichment results. Immunofluorescence analysis revealed that inflammasome-related proteins NLRP3 and IL-1β were significantly increased in recurrent tissues compared with their paired primary samples (Fig. 4A–C), whereas epithelial barrier markers occludin and E-cadherin were markedly decreased (Fig. 4A, D-E). These findings were further validated in the prospective cohort, where RT-qPCR analysis consistently demonstrated elevated mRNA levels of NLRP3 and IL-1β, along with reduced occludin and E-cadherin expression, in the recurrent group relative to non-recurrent patients and controls (Fig. 5A–D). Correlation analysis revealed that P2X7 mRNA levels were positively correlated with NLRP3 and IL-1β, and negatively correlated with occludin and E-cadherin (Fig. 5E–H). Collectively, these data indicate that P2X7 upregulation is closely associated with concurrent inflammasome activation and epithelial barrier disruption in recurrent CRSwNP.

### P2X7 mediates ATP-induced inflammasome activation and barrier dysfunction

**Fig. 6:**
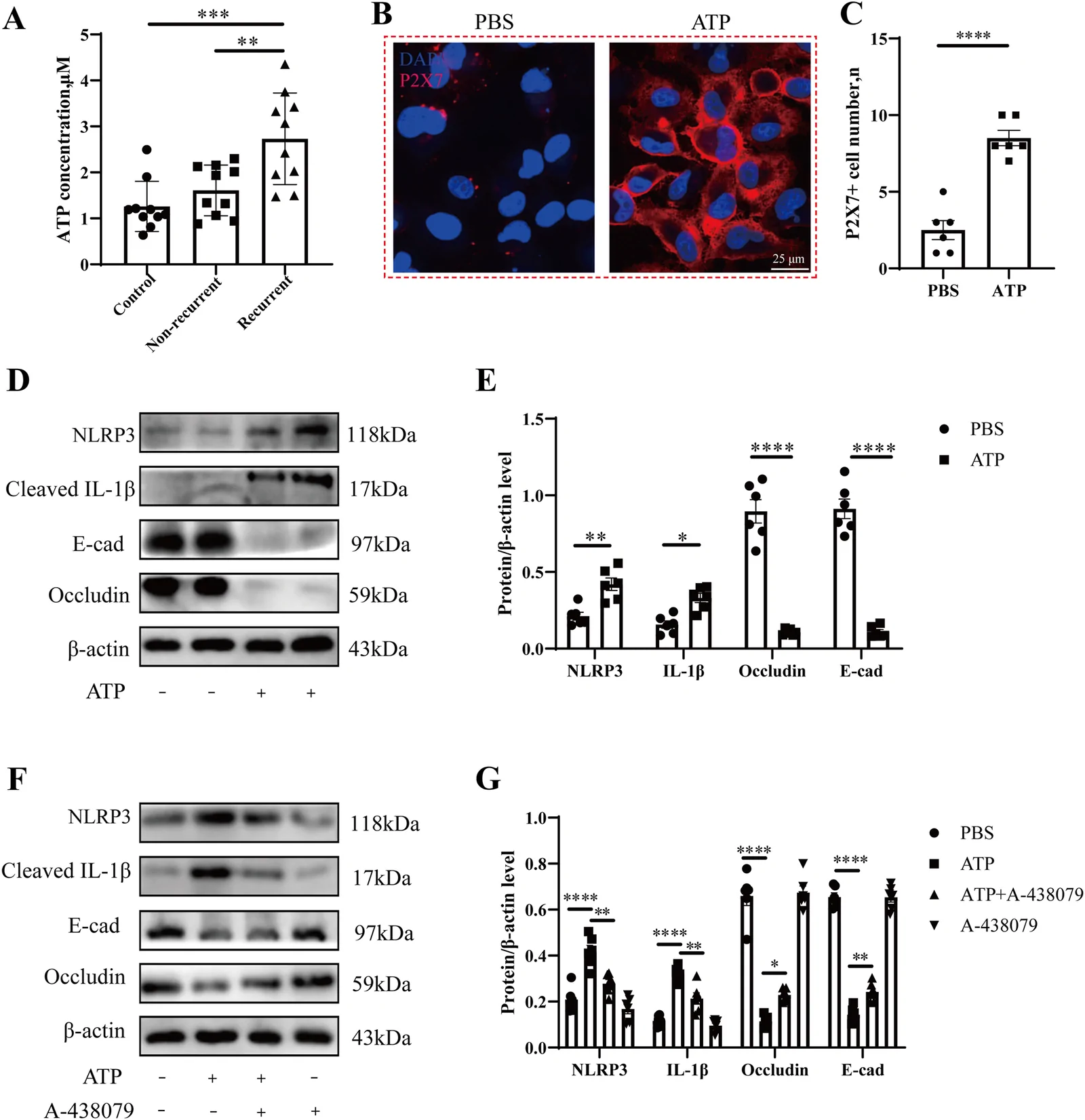
**P2X7 mediates ATP-induced inflammasome activation and barrier dysfunction.** (A) ATP concentrations in control, primary, and recurrent tissue samples. (B–C) P2X7 immunofluorescence images of cells treated with different agents. (D–E) Western blot and quantitative analysis of NLRP3, cleaved IL-1β, E-cad, and occludin in cells treated with PBS or ATP. (F–G) Western blot and quantitative analysis of selected proteins in cells treated with different conditions. ∗P < 0.05, ∗∗P < 0.01, ∗∗∗P < 0.001, ∗∗∗∗P < 0.0001

Given that P2X7 functions as an ATP-gated ion channel, we next examined whether its endogenous ligand, ATP, was altered in recurrent CRSwNP. Notably, ATP levels were significantly elevated in recurrent tissues compared with non-recurrent and control groups (Fig. 6A). In primary human NECs, immunofluorescence staining revealed that ATP stimulation directly upregulated P2X7 expression (Fig. 6B–C). WB analysis further demonstrated that ATP treatment significantly increased the protein levels of NLRP3 and cleaved IL-1β, while reduced occludin and E-cadherin expression (Fig. 6D–E). To determine whether these ATP-induced effects were mediated through P2X7, cells were co-treated with the specific P2X7 antagonist A-438079. Remarkably, pharmacological blockade of P2X7 effectively reversed the ATP-induced molecular alterations, attenuating the upregulation of NLRP3 and cleaved IL-1β and restoring occludin and E-cadherin expression (Fig. 6F–G). Collectively, these findings indicate that the ATP/P2X7 axis plays a critical role in promoting inflammasome activation and epithelial barrier disruption in recurrent CRSwNP.

## Discussion

In this study, paired tissue proteomics revealed substantial protein alterations between pre- and post-recurrence nasal polyps. Among the top upregulated candidates, P2X7 was validated as the only molecule with robust predictive value for postoperative recurrence. Mechanistically, ATP accumulation in recurrent tissues activated P2X7, leading to NLRP3 inflammasome activation and epithelial barrier disruption, effects reversed by P2X7 antagonism.

Several proteomic studies have been conducted in CRSwNP using intraoperative biopsies or nasal lavage fluid.^23^^,^^24^ Notably, Gao et al^25^ identified serum CD163 and TGF-β1 as recurrence-associated proteins, while LOXL2 was recently reported to promote epithelial-mesenchymal transition in eosinophilic CRSwNP.^26^ To our knowledge, none of these previous studies used paired pre- and post-recurrence tissue samples from the same patients, nor did they identify P2X7 as a recurrence-related protein. Apart from proteomic markers, tissue/blood eosinophil counts and serum IgE levels are well-established non-proteomic predictors of CRSwNP recurrence.^27^, ^28^, ^29^ Compared to these conventional markers, which mainly reflect type 2 inflammation, our findings highlight P2X7 as an upstream regulator of inflammasome activation and barrier dysfunction, potentially offering independent predictive information. Our study therefore offers a novel longitudinal perspective on dynamic protein changes in recurrent CRSwNP.

P2X7, a member of the purinergic receptor family, is widely distributed across various tissues, serving as a master regulator of inflammatory responses.^30^, ^31^, ^32^ Previous studies have implicated P2X7 in airway inflammatory diseases, including asthma, where it contributes to airway inflammation and hyperresponsiveness.^33^^,^^34^ However, its specific expression profile and functional role in CRSwNP recurrence remained largely unexplored. Our proteomic screening and subsequent validation consistently demonstrated that P2X7 is significantly upregulated in recurrent CRSwNP tissues, with predominant localization to the nasal epithelium. Importantly, ROC and Kaplan-Meier analyses established P2X7 as a reliable prognostic indicator for early postoperative relapse, providing a strong clinical rationale for further mechanistic investigation.

Given that P2X7 functions exclusively as an ATP-gated ion channel, its upregulation inherently points to the involvement of its endogenous ligand.^30^^,^^35^^,^^36^ We observed significantly elevated ATP levels in recurrent CRSwNP tissues, and *in vitro* experiments confirmed that ATP stimulation directly upregulated P2X7 expression in primary nasal epithelial cells. Although ATP accumulation and subsequent purinergic signaling have been implicated in various inflammatory airway diseases.^37^^,^^38^ Zhang et al^39^ found that the level of airway ATP in asthmatic patients increased, and was highly correlated with the expression of T2 cytokines. However, the specific role of ATP in the recurrence of CRSwNP has not yet been clarified.

Bioinformatics analysis directed our attention to the NOD-like receptor signaling pathway and tight junction integrity, both of which are closely associated with inflammation and barrier disruption. Previous study showed that the ATP/P2X7 axis could trigger intestinal inflammation and promote fibrosis through NLRP3 inflammasome activation.^40^ In additional, inhibition of ATP/P2X7-NLRP3 inflammatory axis could reduce cardiac inflammation and fibrosis and improve cardiac dysfunction.^22^ Moreover, Li et al^41^ found that ATP/P2X7-NLRP3 axis participated in mediating airway inflammation and promoted Th2 inflammatory responses in asthmatic mice. In CRSwNP, NLRP3 inflammasome signaling has been reported to play a pro-inflammatory role and correlate with eosinophilia.^42^, ^43^, ^44^, ^45^ Emerging evidence also highlights eosinophilic inflammation and epithelial barrier dysfunction as key pathological features of recurrent CRSwNP.^3^^,^^46^^,^^47^ In line with these observations, our study demonstrated that recurrent tissues exhibited significantly elevated levels of NLRP3 and IL-1β, along with reduced occludin and E-cadherin expression. Correlation analyses further revealed that P2X7 expression was positively associated with inflammasome activation and negatively associated with barrier integrity. Importantly, ATP stimulation induced upregulation of NLRP3 and cleaved IL-1β, while downregulating occludin and E-cadherin in nasal epithelial cells, and these effects were completely reversed by a specific P2X7 antagonist. Collectively, these results indicate that the ATP/P2X7 axis contributes to CRSwNP recurrence by driving inflammasome activation and disrupting the epithelial barrier.

Several limitations of this study should be acknowledged. First, as a proteomics-based discovery study, our approach has inherent technical limitations, including limited protein coverage, potential quantification bias, and lack of independent validation. Moreover, the small sample size for paired proteomic analysis may compromise statistical power and limit generalizability. Second, although we identified ATP accumulation as a key driver of P2X7 activation, the upstream sources of sustained ATP release in recurrent nasal mucosa require further investigation. Third, while our *in vitro* experiments elucidated the ATP/P2X7/inflammasome axis, *in vivo* validation in appropriate animal models is still needed. Fourth, although our prospective cohort validated the association between P2X7 expression and recurrence, large-scale, multicenter studies with control group are necessary to establish standardized clinical thresholds for P2X7 as a routine predictive biomarker. Lastly, this study cannot completely rule out the possibility of synergistic effects from immune cells in the submucosal layer, particularly P2X7 expressed by mast cells, driving tissue remodeling, which requires further investigation.

## Conclusion

This study provides the first paired proteomic characterization of pre- and post-recurrence CRSwNP tissues, identifying P2X7 as a key upregulated molecule with strong prognostic value. Mechanistically, we demonstrate that ATP/P2X7 signaling participates in recurrence by activating the NLRP3 inflammasome and disrupting epithelial barrier integrity. These findings not only advance our understanding of the molecular underpinnings of CRSwNP recurrence but also offer a potential biomarker and therapeutic target for preventing early postoperative relapse.

## Availability of data and material

All data generated or analyzed in this study are contained in this article and its supplement. Additional data are available from the corresponding author upon reasonable request.

## Author contribution

(I) Conception and design: Linfei Yang and Qiong Liu.

(II) Administrative support: Linfei Yang and Qiong Liu.

(III) Sample collection and data analysis: Linfei Yang, Yi Chen, Ting Shi.

(IV) Histopathological and cellular experiments: Linfei Yang and Yi Chen.

(V) Manuscript writing: All authors.

(VI) Final approval of manuscript: All authors.

## Ethics approval

This study was approved by the Human Ethics Committee of Hunan Provincial People's Hospital (The First-Affiliated Hospital of Hunan Normal University) (2,026,036), and all participants provided written informed consent in accordance with the Declaration of Helsinki.

## Consent for publication

All authors have seen and approved the last version and agreed to the publication of the work.

## Disclosure statement

No generative AI and AI-assisted technologies were used. Nothing to disclose.

## Funding source

This research was supported by the 10.13039/501100004735Natural Science Foundation of Hunan Province (Grants 2026JJ82047) and the Renshu Fund of 10.13039/501100017545Hunan Provincial People's Hospital (No.RS201904).

## Declaration of competing interest

There are no patents, products in development, or marketed products to declare.
