# Supplementary material for: ATP/P2X7 signaling as a potential predictive biomarker for early recurrence of nasal polyps: Correlation with inflammasome activation and epithelial barrier dysfunction
Source: World Allergy Organ J. 2026 Aug 28;19(9):101455. doi: 10.1016/j.waojou.2026.101455 (PMC13546894; doi:10.1016/j.waojou.2026.101455)
Supplement: Supplementary file 1 [file mmc1.docx]

Figure S1.

Figure S1. Isotype controls were performed on control nasal mucosal sections as a technical negative control to verify antibody specificity.
