# Supplementary material for: ATP/P2X7 signaling as a potential predictive biomarker for early recurrence of nasal polyps: Correlation with inflammasome activation and epithelial barrier dysfunction
Source: World Allergy Organ J. 2026 Aug 28;19(9):101455. doi: 10.1016/j.waojou.2026.101455 (PMC13546894; doi:10.1016/j.waojou.2026.101455)
Supplement: Supplementary file 2 [file mmc2.docx]

Table S1. Primer sequences

| Gene | Forward primer | Reverse primer |
| --- | --- | --- |
| *GAPDH* | ACGCTGCAGGGCCTCACTCCTTTT | TCCGACGCCTGCTTCACCACCTTCT |
| *P2X7* | TGGTAAGTGGGATCTGGGGAGGACC | TGTGGCTTCTCTCTGCTGCAGCCT |
| *PDE4D* | ACAGTGCTGAGAGTGAAGCCCT | TGTGATCGTTTCAGGCTTCCCAGC |
| *JADE2* | TCAAGTCGGGAAGTGGAGCC | AGAAGGCAGTGATGAGCGGGTGGGT |
| *NLRP3* | AGGCTTCCTAGTCCCCCTAAGACT | TCCACAGGCTAGGCACTCACTGA |
| *IL-1β* | ACGTAGCCGTCATGGGGAAGTCA | TGGTCTCAGCGTCTCCACTGACCT |
| *Occludin* | AGAGCAGCACCGGCCAACTT | AGTGAAAAGTTGCCCGCGCCGCTGA |
| *E-cadherin* | TGACGCCGAGAGCTACACGTTCACG | TGCGTAAATTCCAAGGGGTGTCGT |
